# Supplementary material for: Somatic NAP1L1 p.D349E promotes cardiac hypertrophy through cGAS-STING-IFN signaling
Source: Nat Commun. 2025 Apr 1;16:3140. doi: 10.1038/s41467-025-58453-7 (PMC11961713; doi:10.1038/s41467-025-58453-7)
Supplement: Supplementary file 1 — Supplementary Information [file 41467_2025_58453_MOESM1_ESM.pdf]

Supplementary Table S1. Baseline information of the replication cohort

	Gene-elusive Patients	Patients with somatic NAP1L1 p.D349E	p value
Number of Patients	37	12	
Male, n	21	7	0.87
Age at diagnosis (years)	38±13.63	42±10.56	0.58
Duration of disease (years)	6.7±3.8	7.4±2.6	0.68
BMI (kg/m2)	21.3±2.7	20.6±3.9	0.83
Co-morbidities			
Diabetes, n	8	4	0.72
Hypercholesterolemia, n	5	2	0.64
Atrial Fibrillation, n	2	0	0.16
Heart Rate, n	80.4±7.2	77.4±6.8	0.24
Medications			
Beta blockers, n	24	8	0.74
Calcium channel blockers, n	13	4	0.49
Max. LV Thickness (mm)	23.40±4.78	21.64±3.76	0.52
LVEF (%)	64.38±5.17	65.53±3.94	0.19
LV End Diastolic Dimension (mm)	48.35±6.37	46.54±2.07	0.27
NYHA class II	29	1	
NYHA class III	7	8	
NYHA class IV	1	3	

Supplementary Table S2. ddPCR Sensitivity and Accuracy for Low-Frequency Variant Detection

Real VAF (%)	Fam	Fam+Hex	VAF by ddCPR (%)
0.03	3	10260	0.0292
0.04	4	10740	0.0372
0.05	5	10380	0.0482
0.10	13	12240	0.1062
0.50	57	11890	0.4794
1.00	125	12620	0.9905
2.00	238	11940	1.9933
5.00	596	11860	5.0253
10.00	1026	10280	9.9805

Supplementary Table S3. Patients carrying somatic *NAP1L1* D349E

Patient ID	Maximal LV wall thickness(mm)	VAF (%)	Identified by
D6	35	4.00	WES+ddPCR
D11	36	1.26	ddPCR
D26	32	0.87	ddPCR
D45	19	0.24	ddPCR
D47	22	2.08	WES+ddPCR
D55	30	0.45	ddPCR
D60	32	0.16	ddPCR
R3	25	2.11	ddPCR
R7	26	0.31	ddPCR
R9	24	0.61	ddPCR
R16	25	1.52	ddPCR
R18	30	0.91	ddPCR
R22	23	1.08	ddPCR
R26	38	0.77	ddPCR
R31	21	0.53	ddPCR
R35	27	2.31	ddPCR
R38	29	3.04	ddPCR
R42	27	0.87	ddPCR
R48	30	0.62	ddPCR

D6 to D60 represent seven patients in the discovery cohort, and R3 to R48 represent twelve patients in the replication cohort.

Supplementary Table S4. 58 genes related to HCM

ABCC9	*CALR3*	*FLNC*	*LZTR1*	*MYOZ2*	*RIT1*	*TRIM63*
ACTA1	*CASQ2*	*FXN*	*MYBP3*	*MYPN*	*RYR2*	*TTN*
ACTC1	*CAV3*	*GAA*	*MYH6*	*NEXN*	*SLC254*	*TTR*
ACTN2	*COX15*	*GLA*	*MYH7*	*OBSCN*	*TCAP*	*VCL*
ALPK3	*CRYAB*	*JPH2*	*MYL2*	*PDLIM3*	*TMPO*	
ANKR1	*CSRP3*	*KCNQ1*	*MYL3*	*PLN*	*TNNC1*	
BAG3	*DES*	*KLF10*	*MYLK2*	*PRKA2*	*TNNI3*	
CACN1C	*DSP*	*LAMP2*	*MYO6*	*PTPN11*	*TNNT2*	
CACN2	*FHL1*	*LDB3*	*MYOM1*	*RAF1*	*TPM1*	

Supplementary Figure S1. Workflow of the discovery cohort.

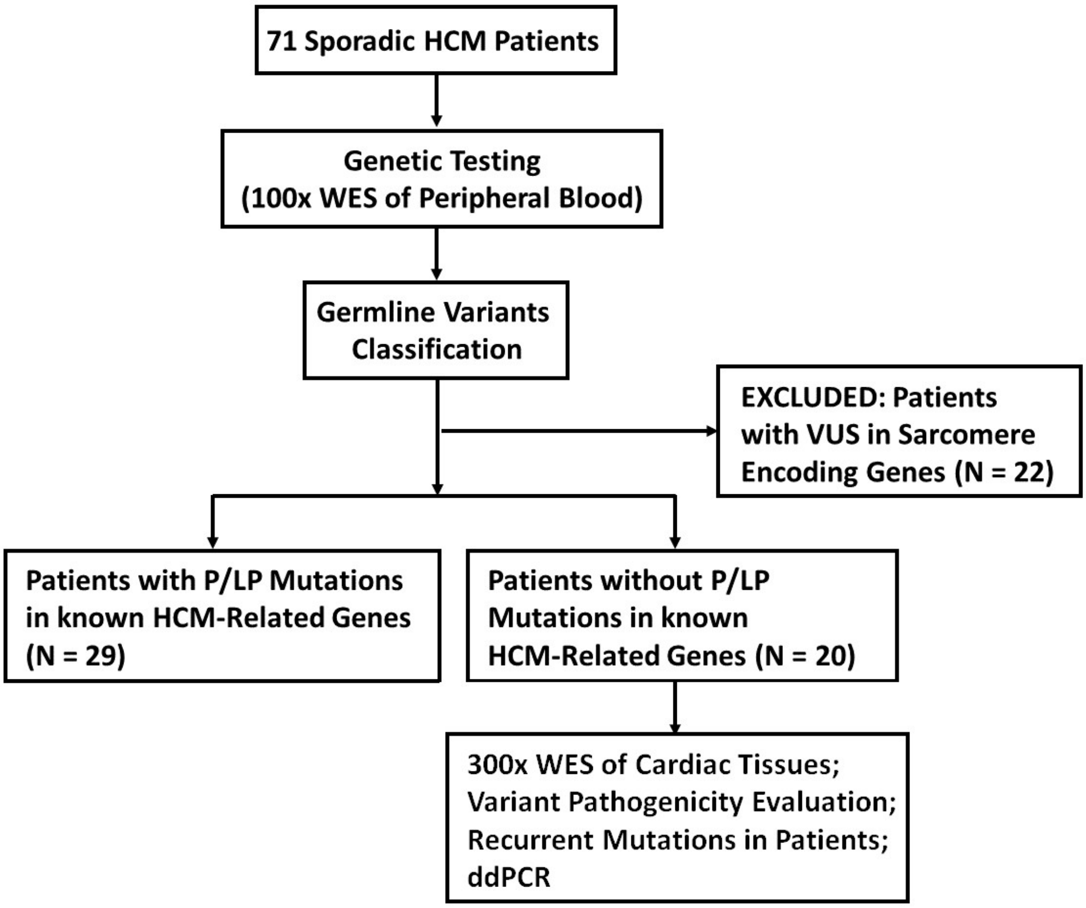

Figure legend: Flow diagram of study participants in the discovery cohort. Abbreviations: HCM, hypertrophic cardiomyopathy; P/LP variants, pathogenic/likely pathogenic variants; VUS, variants of uncertain significance; WES, whole exome sequencing.

Supplementary Figure S2. Representative negative ddPCR plots and validation of ddPCR

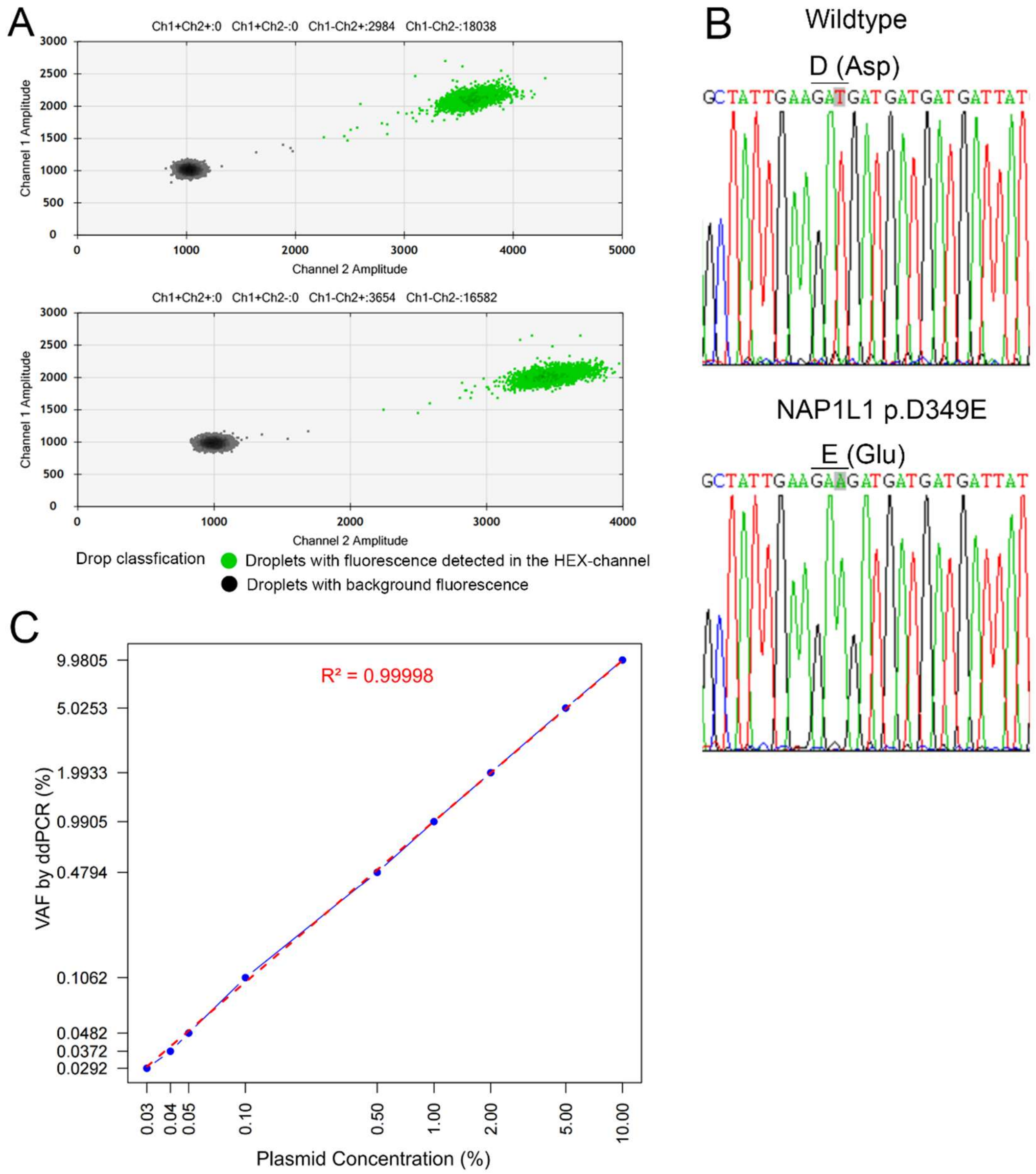

Figure legends: A, *Upper*, plot of ddPCR result on negative control H7; *Lower*, plot of ddPCR result on negative control H14; **B**, Validation of NAP1L1 p.D349E. PCR amplicons were cloned and subjected to Sanger sequencing analysis. The sequencing electrophoretograms are shown (*Left*, wild-type allele; *Right*, mutant allele); **C,** Correlation curve of observed VAFs (y-axis) versus expected VAFs (x-axis) for each plasmid mixture. Plasmid mixtures containing the target somatic variant at defined

variant allele frequencies (VAFs) ranging from 0.03% to 10% were analyzed by ddPCR.

Supplementary Figure S3. IGV of NAP1L1 p.D349E.

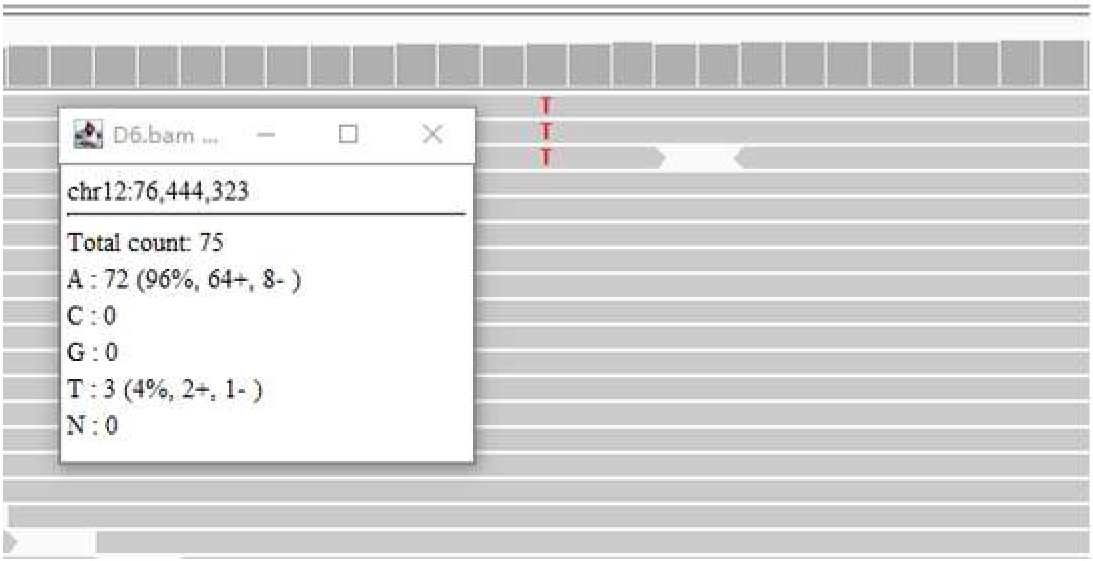

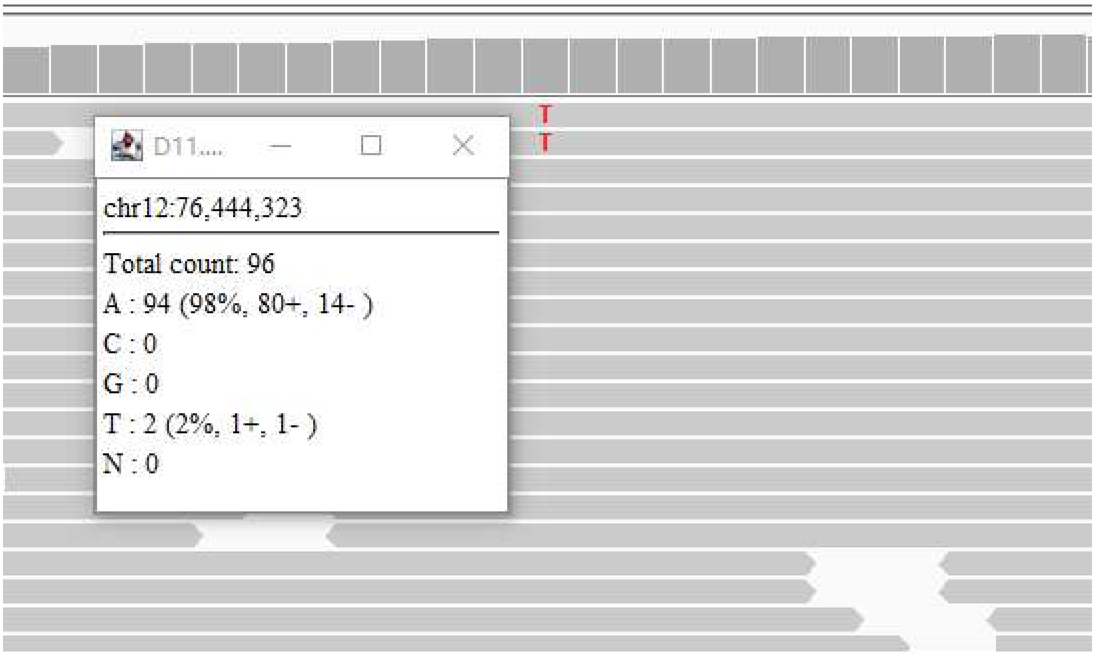

Figure legend: Screenshot of somatic variant *NAP1L1* D349E in raw aligned sequence data viewed by integrative genome viewer**.**

Supplementary Figure S4. The fractional abundance of NAP1L1 p.D349E in cTnT+ and cTnT− cells

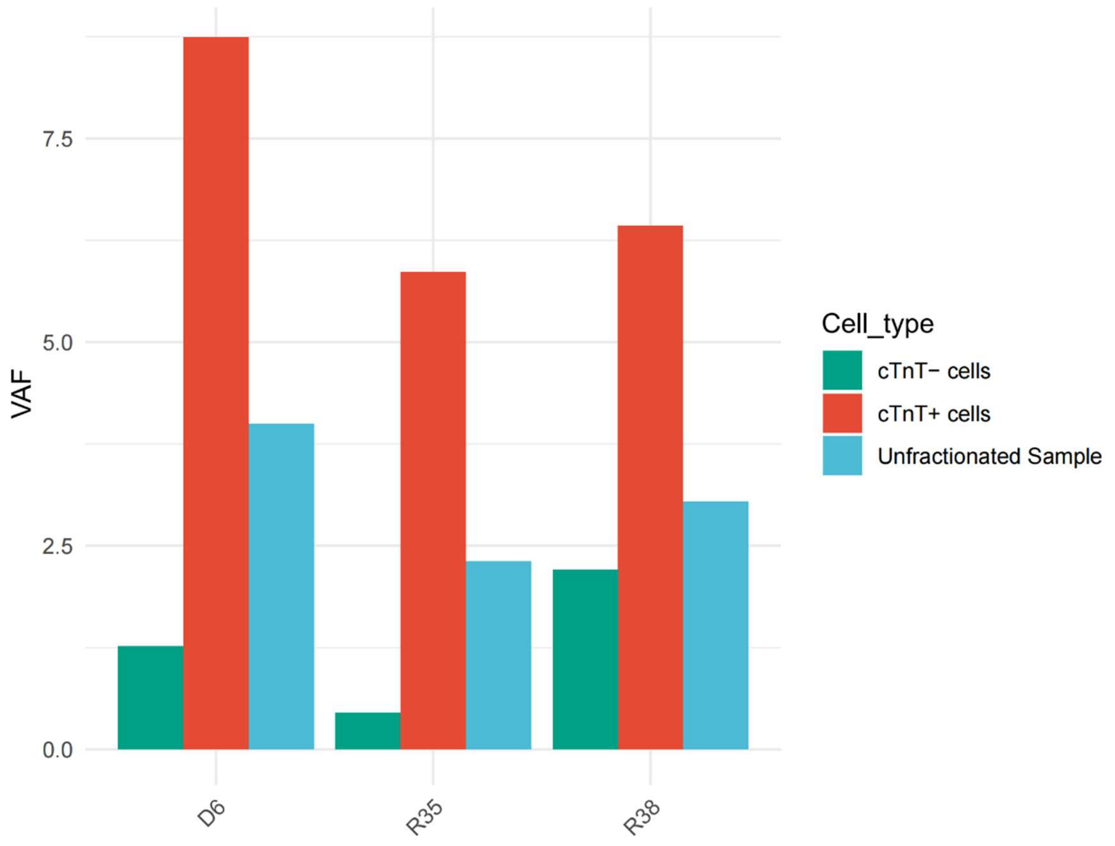

Figure legend: The fractional abundance of NAP1L1 p.D349E in cTnT+ and cTnT− cells from patients P6, R35, and R38, as well as in the unfractionated whole tissue sample from an adjacent tissue section.

Supplementary Figure S5. Knockdown of *NAP1L1* triggered the cytosolic DNA-sensing pathway

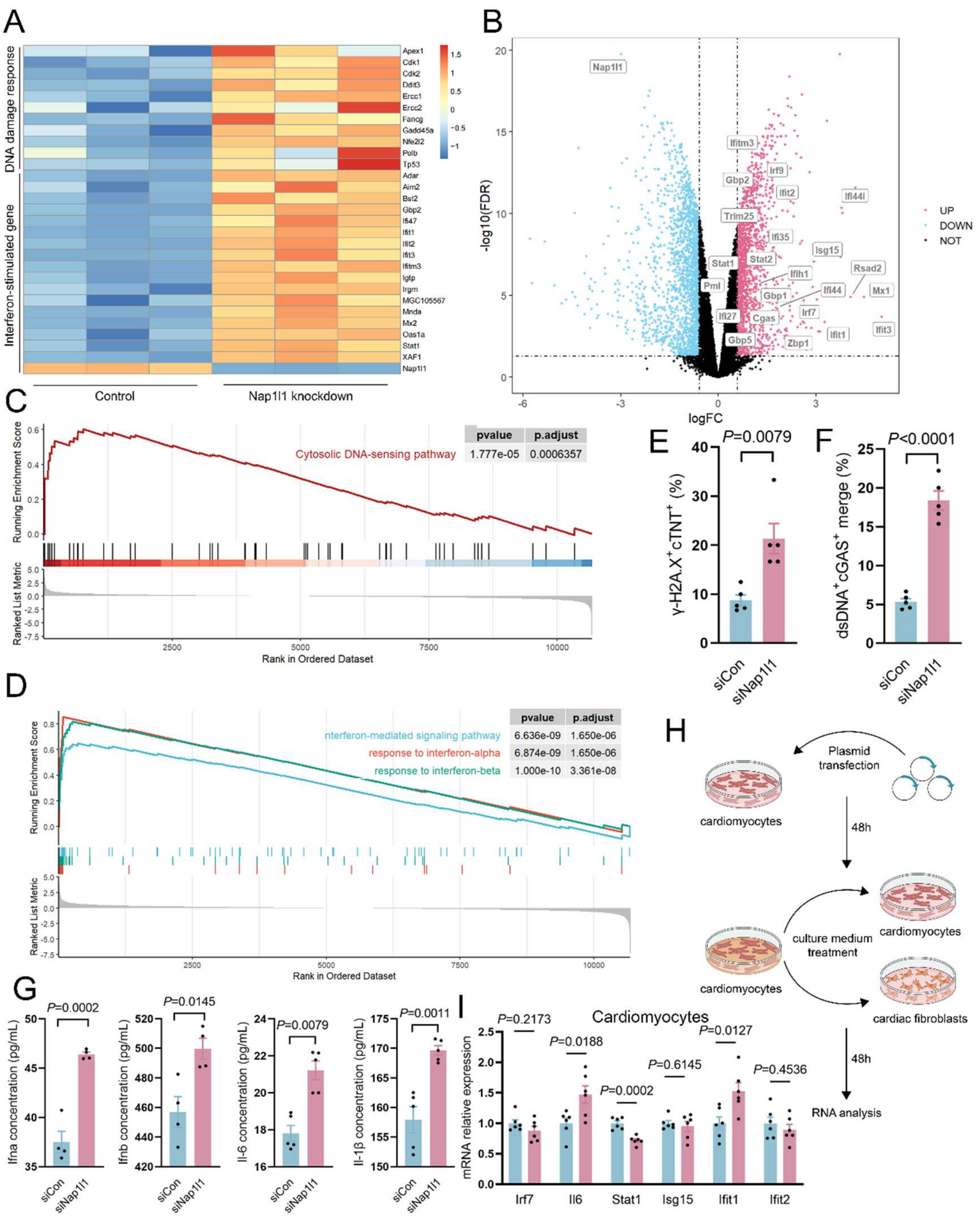

Figure legends: A, Heatmap of RNA-seq and upregulated interferon-stimulated genes and Cgas. **B**, Volcano plot of interferon-stimulated genes. **C,** GSEA plot of the upregulated cytosolic DNA-sensing pathway in the *Nap1l1* knockdown group. **D**, GSEA plot of upregulated interferon response pathways in the *Nap1l1* knockdown

group. **E,** Quantification of γ-H2-A.X+ area in cardiomyocytes. N = 5 for each group and two-tailed unpaired Student's t test was applied; **F,** Quantification of dsDNA+ and cGAS+ area. N = 5 for each group and two-tailed unpaired Student's t test was applied; **G,** Elisa of Ifna, Ifnb, Il6 and Il-lβ in Nap1l1 knockdown compared with control group. N = 5 for each group and two-tailed unpaired Student's t test was applied; **H,** Schematic diagram of in vitro simulating the effects of cardiomyocytes and cardiac fibroblasts. Created in BioRender. Lv, C. (2025) https://BioRender.com/v66i322 ; **I,** qPCR analysis of Irf7, Il6, Stat1, Isg15, Ifit1 and Ifit2 in influenced cardiomyocytes. N = 6 for each group and two-tailed unpaired Student's t test was applied.

Supplementary Figure S6. Nap1l1 p.D349E in vivo promoted cardiac hypertrophy.

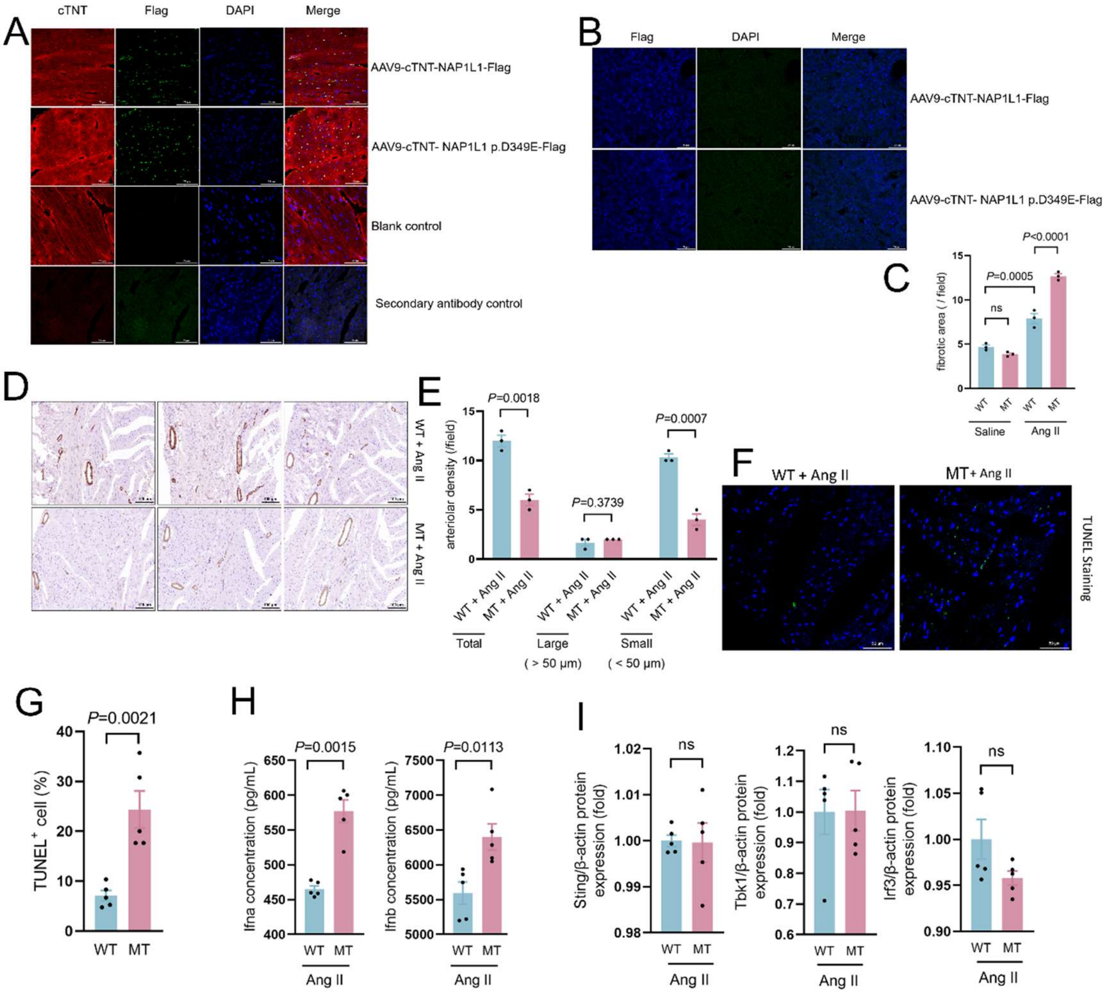

Figure legends: A, Immunofluorescence staining of cTnT, Flag and DAPI in cardiac tissues; **B,** Immunofluorescence staining of DAPI and Flag in renal parenchymal cells; **C,** Quantification of fibrotic area in WT or MT group with Saline or Ang II. N = 3 for each group and one-way ANOVA with Tukey tests was used for correction of multiple comparisons was applied; **D,** F4/80 staining showing the macrophage infiltration; **E,** Quantification of the density of total and small arteriolar area. N = 3 for each group and two-tailed unpaired Student's t test was applied; **F,** TUNEL staining showing the level of apoptosis. **G,** Quantification of TUNEL+ area. N = 5 for each group and two-tailed unpaired Student's t test was applied; **H,** Elisa of Ifna, Ifnb of the left ventricle tissues WT or MT group with Ang II. N = 5 for each group and two-tailed unpaired Student's t test was applied; **I,** Quantification of total Sting, Tbk1 and Irf3 expression in the WT with Ang II and MT with Ang II groups. N = 5 for each group and two-tailed unpaired

Student's t test was applied.

Supplementary Figure S7. Nap1l1 p.D349E knock-in (KI) mice.

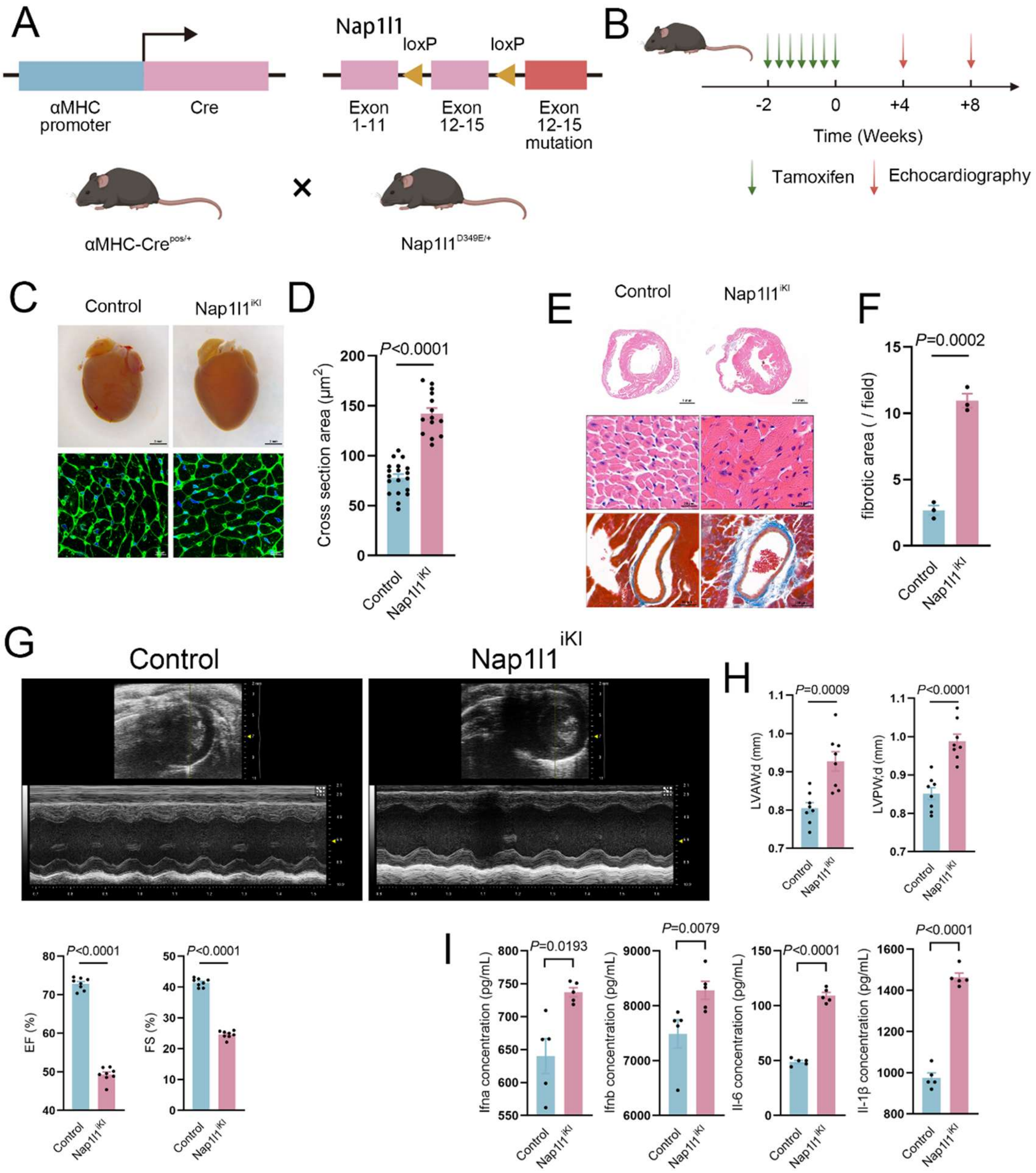

Figure legends: A and **B**, Schematic diagram depicting the experimental strategy used in C57BL6/N mice. Created in BioRender. Lv, C. (2025) https://BioRender.com/v66i322 **C**, Representative image of heart and WGA staining; **D,** Quantification of WGA staining in the control and KI group. N = 19 for the control and N = 14 for Nap1l1^{iKI} and two-tailed unpaired Student's t test was applied; **E**, Representative hematoxylin and eosin staining Masson's trichrome staining; **F,** Quantification showing the fibrotic area in control and KI group. N = 3 for each group and two-tailed unpaired Student's t test was applied; **G**, Representative M-mode

echocardiograms of the left ventricle of the control and KI group. **H**, Quantification of left ventricular end-diastolic anterior wall thickness (LVAW; d), left ventricular end-diastolic posterior wall thickness (LVPW; d) ejection fraction (EF), fractional shortening (FS) of the control and KI group. N = 8 for each group and two-tailed unpaired Student's t test was applied; **I,** Elisa of Ifna, Ifnb, Il6 and Il-lβ of the left ventricle tissues of the control and KI group. N = 5 for each group. for Ifna, two-tailed Unpaired t test with Welch's correction, for Ifnb, two-tailed Unpaired Mann-Whitney U test and for Il-6 and Il-1b, two-tailed unpaired Student's t test were applied.

Supplementary Figure S8. Nap1l1 p.D349E triggered cGAS-Sting-IFN signaling.

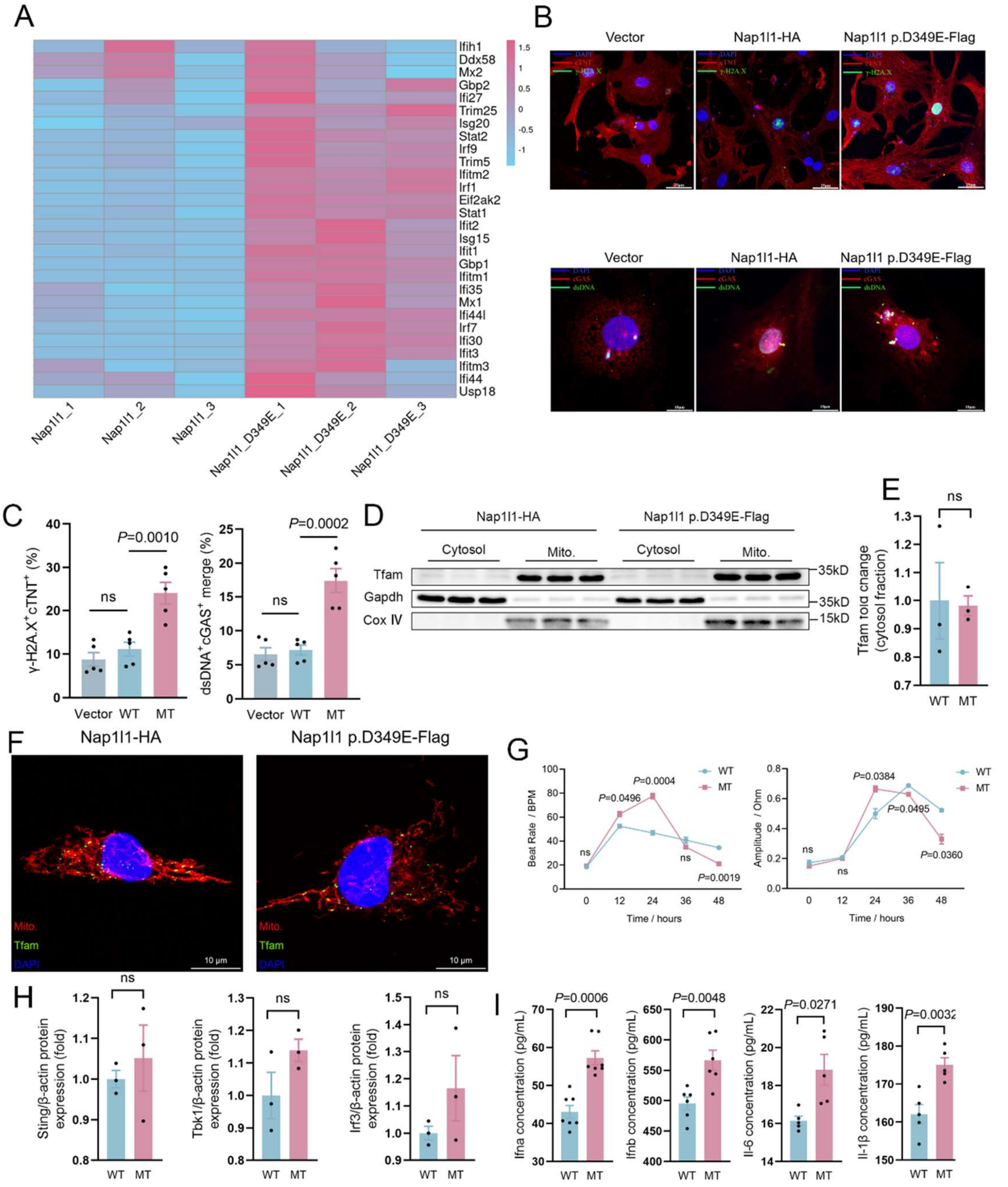

Figure legends: A, Heatmap of the expression of interferon-stimulated genes in vector, WT and MT groups; **B and C**, γ -H2-A.X staining and dsDNA staining and quantification in Vector, WT and MT groups. N = 5 and one-way ANOVA with Tukey tests was used for correction of multiple comparisons; **D and E,** Western blot image and quantification of Tfam in the cytosol and mitochondria. N=3 and two-tailed

unpaired Student's t test were applied; **F,** Mito., Tfam and DAPI staining in WT and MT groups; **G,** Measurement of heart rate and diastolic function over time in WT or MT groups**.** N = 4 and one-way ANOVA with Tukey tests was used for correction of multiple comparisons; **H,** Quantitative of Sting, Tbk1 and Irf3 in the WT and MT groups. N=3 and two-tailed unpaired Student's t test were applied; **I,** Elisa of Ifna, Ifnb, Il6 and Il-lb in the WT and MT groups. N = 7, 6, 5 and 5 for Ifna, Ifnb, Il-6 and Il-1b, and two-tailed unpaired Student's t test were applied;

Supplementary Figure S9. The safety of pharmacological inhibition of cGAS-STING-IFN signaling

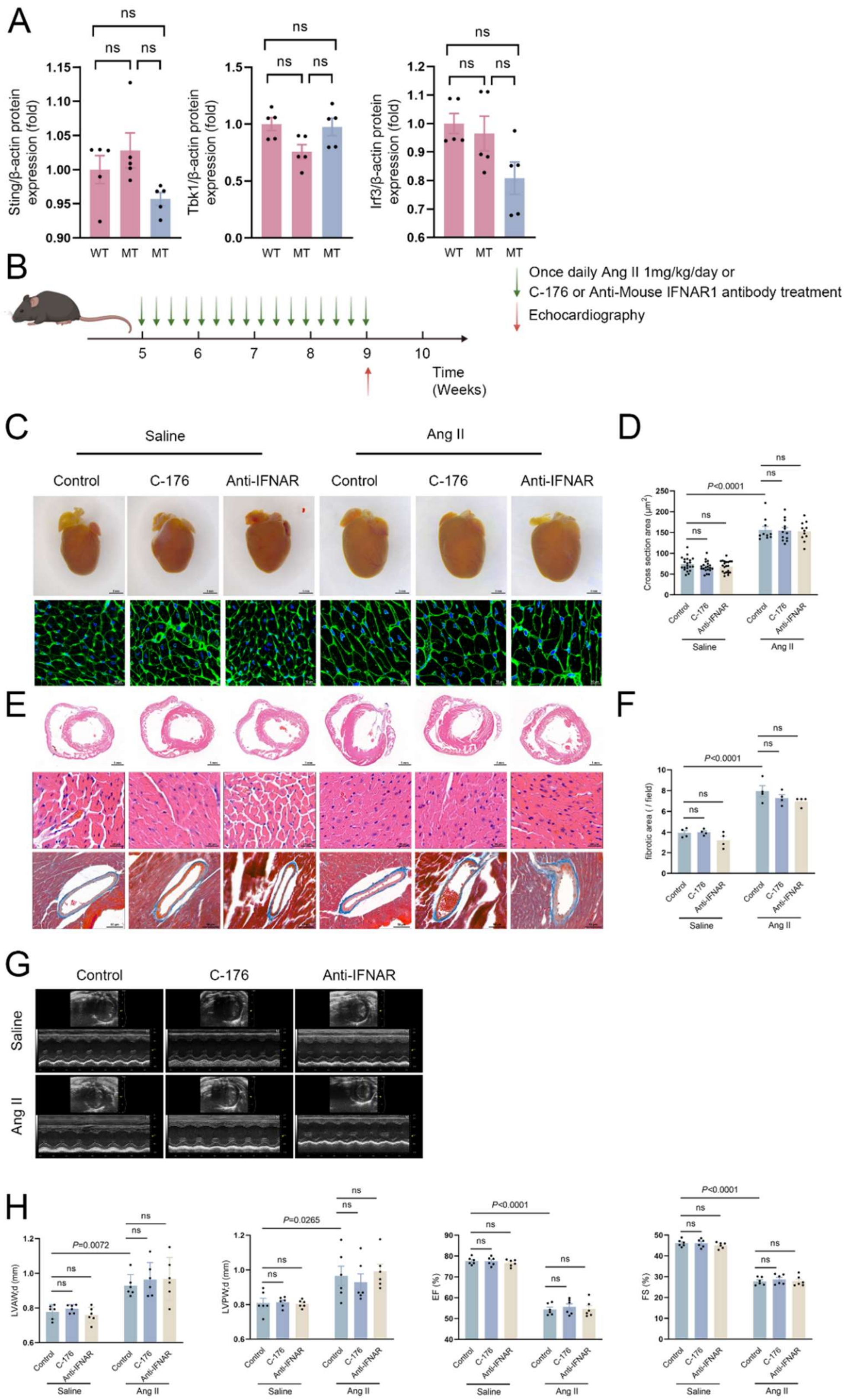

Figure legends: A, Quantitative of Sting, Tbk1 and Irf3 in the corresponding groups. N = 5 for each group and one-way ANOVA with Tukey tests was used for correction of multiple comparisons; **B**, Schematic diagram depicting the experimental strategy used in C57BL6/N mice. Created in BioRender. Lv, C. (2025) https://BioRender.com/v66i322. **C** and **D**, Representative image of heart and wheat germ agglutinin (WGA) staining and quantification of the WT mice treated with C-176 and anti-mouse Ifnar1 antibodies at baseline or exposed to angiotensin II. In Saline group, N = 21 for control, 20 for C-176 and 21 for anti-mouse Ifnar1. In Ang II group, N = 10 for control, 12 for C-176 and 11 for anti-mouse Ifnar1. one-way ANOVA with Tukey tests was used for correction of multiple comparisons was applied; **E** and **F**, Representative hematoxylin and eosin staining Masson's trichrome staining and quantification showing the fibrotic area of the WT mice treated with C-176 and anti-mouse Ifnar1 antibodies at baseline or exposed to angiotensin II. N = 4 for each group and one-way ANOVA with Tukey tests was used for correction of multiple comparisons; **G**, Representative M-mode echocardiograms of the left ventricle of the WT mice treated with C-176 and anti-mouse IFNAR1 antibodies at baseline or exposed to angiotensin II. **H**, Quantification of left ventricular end-diastolic anterior wall thickness (LVAW; d), left ventricular end-diastolic posterior wall thickness (LVPW; d), ejection fraction (EF), fractional shortening (FS). N = 6 for each group and one-way ANOVA with Tukey tests was used for correction of multiple comparisons;
