# Supplementary material for: Somatic NAP1L1 p.D349E promotes cardiac hypertrophy through cGAS-STING-IFN signaling
Source: Nat Commun. 2025 Apr 1;16:3140. doi: 10.1038/s41467-025-58453-7 (PMC11961713; doi:10.1038/s41467-025-58453-7)
Supplement: Supplementary file 2 — Description of Additional Supplementary Information [file 41467_2025_58453_MOESM2_ESM.pdf]

Supplementary Data1.
Description: P/LP germline variants identified in patients with HCM
Abbreviations: LP, likely pathogenic; P, pathogenic.
Supplementary Data 2.
Description: VUS identified in patients with HCM
Abbreviations: VUS, Variants of unknown significance
Supplementary Data 3.
Description: NAP1L1 c1047 among 100 vertebrate species
Supplementary Data 4.
Description: Primary antibodies used in this study.
Supplementary Data 5.
Description: Sequences of Primers used in this study.
