# Supplementary material for: Somatic NAP1L1 p.D349E promotes cardiac hypertrophy through cGAS-STING-IFN signaling
Source: Nat Commun. 2025 Apr 1;16:3140. doi: 10.1038/s41467-025-58453-7 (PMC11961713; doi:10.1038/s41467-025-58453-7)
Supplement: Supplementary file 8 — Reporting Summary [file 41467_2025_58453_MOESM8_ESM.pdf]

nature portfolio

Corresponding author(s): Yibo Wang

Last updated by author(s): Jan 14, 2025

Reporting Summary

Nature Portfolio wishes to improve the reproducibility of the work that we publish. This form provides structure for consistency and transparency in reporting. For further information on Nature Portfolio policies, see our Editorial Policies and the Editorial Policy Checklist.

Statistics

For all statistical analyses, confirm that the following items are present in the figure legend, table legend, main text, or Methods section.

n/a	Confirmed	
☐	☒	The exact sample size (n) for each experimental group/condition, given as a discrete number and unit of measurement
☐	☒	A statement on whether measurements were taken from distinct samples or whether the same sample was measured repeatedly
☐	☒	The statistical test(s) used AND whether they are one- or two-sided<br>*Only common tests should be described solely by name; describe more complex techniques in the Methods section.*
☐	☒	A description of all covariates tested
☐	☒	A description of any assumptions or corrections, such as tests of normality and adjustment for multiple comparisons
☐	☒	A full description of the statistical parameters including central tendency (e.g. means) or other basic estimates (e.g. regression coefficient) AND variation (e.g. standard deviation) or associated estimates of uncertainty (e.g. confidence intervals)
☐	☒	For null hypothesis testing, the test statistic (e.g. F, t, r) with confidence intervals, effect sizes, degrees of freedom and P value noted<br>*Give P values as exact values whenever suitable.*
☒	☐	For Bayesian analysis, information on the choice of priors and Markov chain Monte Carlo settings
☒	☐	For hierarchical and complex designs, identification of the appropriate level for tests and full reporting of outcomes
☒	☐	Estimates of effect sizes (e.g. Cohen's d, Pearson's r), indicating how they were calculated

Our web collection on statistics for biologists contains articles on many of the points above.

Software and code

Policy information about availability of computer code

Data collection	SP8 Laser Confocal Microscope (Leica)<br>Spectrophotometer (Nanodrop 2000c, Thermo Fisher)<br>Chemiluminescence system (Tanon 5800 Multi, China)<br>VisualSonics Vevo 2100 (VisualSonics, Toronto, Canada)<br>Tanon 5800 Multi (Tanon)<br>QuantStudio 5 (Thermo Fisher Scientific)<br>Pannoramic SCAN (3DHISTECH, Hungary)

Data analysis	Image J software version 2.0.0<br>FlowJo CE(7.5.110.7)<br>GraphPad Prism(version 9.0)and SPSS (version 26.0)<br>R(version 4.1.2)<br>All the replicate experiments (including cell and mouse-based experiments) were biological replicates, which were repeated at least three times. Data are presented as means ± SEM and individual data points are plotted. The normality of the data was tested using the Shapiro–Wilk normality test. The differences between two groups were evaluated using unpaired two-sided Student's t tests, and multiple group comparisons were conducted by two-way ANOVA followed by Bonferroni's multiple comparisons test. A P value < 0.05 was considered statistically significant. Statistical analyses were performed in GraphPad PRISM 9 and SPSS 26.0. The images were created by Adobe Illustrator 2020 software.

For manuscripts utilizing custom algorithms or software that are central to the research but not yet described in published literature, software must be made available to editors and reviewers. We strongly encourage code deposition in a community repository (e.g. GitHub). See the Nature Portfolio guidelines for submitting code & software for further information.

Data

Policy information about availability of data

All manuscripts must include a data availability statement. This statement should provide the following information, where applicable:

- Accession codes, unique identifiers, or web links for publicly available datasets
- A description of any restrictions on data availability
- For clinical datasets or third party data, please ensure that the statement adheres to our policy

The authors confirm that data supporting the findings of this study are available in the manuscript and its supplements. The WES data generated in this study have been deposited in the GSA database under accession code PRJCA033060 [https://ngdc.cncb.ac.cn/]. The raw RNA dataset is available at the National Center for Biotechnology Information's Gene Expression Omnibus Database GSE286501 [https://www.ncbi.nlm.nih.gov/geo/query/acc.cgi?acc= GSE286501]. Source data are provided with this paper.

Research involving human participants, their data, or biological material

Policy information about studies with human participants or human data. See also policy information about sex, gender (identity/presentation), and sexual orientation and race, ethnicity and racism.

Reporting on sex and gender	Seventy-one sporadic HCM patients who needed surgical intervention due to left ventricular outflow tract obstruction were recruited from Fuwai Hospital from 2012 to 2015 as the discovery cohort. A replication cohort comprising forty-nine sporadic genetically unexplained HCM patients was recruited from the First Affiliated Hospital of Xi'an Jiaotong University. The Information of participants is presented in detail in the manuscript and supplementary materials.
Reporting on race, ethnicity, or other socially relevant groupings	All participants are Chinese Han people.
Population characteristics	Seventy-one sporadic HCM patients who needed surgical intervention due to left ventricular outflow tract obstruction were recruited from Fuwai Hospital from 2012 to 2015 as the discovery cohort. A replication cohort comprising forty-nine sporadic genetically unexplained HCM patients was recruited from the First Affiliated Hospital of Xi'an Jiaotong University. All of the patients had outflow tract obstructions and underwent septal myectomy. None of the patients' first-degree relatives had HCM. The diagnosis of HCM was based on echocardiography and defined as unexplained left ventricle hypertrophy (maximum wall thickness ≥ 15 mm). We excluded patients with hypertension, valvular disease, congenital disease, infiltrative cardiomyopathy, or other diseases that could lead to abnormal loading conditions.
Recruitment	Seventy-one sporadic HCM patients who needed surgical intervention due to left ventricular outflow tract obstruction were recruited from Fuwai Hospital from 2012 to 2015 as the discovery cohort. A replication cohort comprising forty-nine sporadic genetically unexplained HCM patients was recruited from the First Affiliated Hospital of Xi'an Jiaotong University. All participants gave their signed informed consent to the study. There was no<br>self-selection bias or any other bias during the recruitment of individuals in this study.
Ethics oversight	The study was reviewed and approved by the ethics committees of Fuwai Hospital and the First Affiliated Hospital of Xi'an Jiaotong University

Note that full information on the approval of the study protocol must also be provided in the manuscript.

Field-specific reporting

Please select the one below that is the best fit for your research. If you are not sure, read the appropriate sections before making your selection.

[x] Life sciences [ ] Behavioural & social sciences [ ] Ecological, evolutionary & environmental sciences

For a reference copy of the document with all sections, see nature.com/documents/nr-reporting-summary-flat.pdf

Life sciences study design

All studies must disclose on these points even when the disclosure is negative.

Sample size	The N number for all experiments, including animal experiments, in vitro experiments and clinical sample studies were listed in the figure legends or in Supplementary Table 1-2. The sample size was chosen based on our prior studies (PMID: 34615377, 34397273)and other previous papers with similar experiments (PMID: 28924165, 25628421, 36158197), which showed sufficient statistical power for in vitro experiments and animal experiments
Data exclusions	No samples or animals were excluded from analyses
Replication	All animal experiments were repeated at least twice and in vitro experiments were repeated at least three times. All results are reproducible and representative data were showed in the figures or supplementary files.
Randomization	Animals were allocated to their respective group at birth by a blinded investigator. For other experiments, including cell experiments, before performing the corresponding treatment, samples were randomly assigned to control and experimental groups by an investigator blinded to subsequent experimental information. The standard laboratory procedures were strictly followed to keeping the experimental environment and facilities consistent and performed under the same conditions.
Blinding	Investigators were blinded to group allocation during data collection, image quantification and data analysis.

Reporting for specific materials, systems and methods

We require information from authors about some types of materials, experimental systems and methods used in many studies. Here, indicate whether each material, system or method listed is relevant to your study. If you are not sure if a list item applies to your research, read the appropriate section before selecting a response.

Materials & experimental systems

n/a	Involved in the study	
☐	☒	Antibodies
☐	☒	Eukaryotic cell lines
☒	☐	Palaeontology and archaeology
☐	☒	Animals and other organisms
☒	☐	Clinical data
☒	☐	Dual use research of concern
☒	☐	Plants

Methods

n/a	Involved in the study	
☒	☐	ChIP-seq
☒	☐	Flow cytometry
☒	☐	MRI-based neuroimaging

Antibodies

Antibodies used

Antibodies Vendor Catalog Working concentration
NAP1L1 proteintech 14898-1-AP WB 1:1000
HA proteintech 66006-2-Ig WB 1:100000
Flag proteintech 66008-4-Ig WB 1:20000
GFP proteintech 50430-2-AP IP 4µg
IgG proteintech 30000-0-AP WB 1:20000
H2A ABclonal A3692 WB 1:1000
H2B Santa Cruz sc-515808 WB 1:1000
Phospho-Histone H2A.X (Ser139) Santa Cruz sc-517348 WB 1:1000
H2A.X proteintech 10856-1-AP WB 1:1000
H2A.Z proteintech 16441-1-AP WB 1:1000
GAPDH proteintech 60004-1-Ig WB 1:100000
Lamin B1 proteintech 66095-1-Ig WB 1:50000
α-SMA ZSGB-BIO ZM-0003 IHC
F4/80 proteintech 28463-1-AP IHC 1:4000
KI-67 Cell Signaling Technology 9129 IF 1:400
β-actin proteintech 81115-1-RR WB 1:20000
cTNT proteintech 15513-1-AP IF 1:250
cGAS proteintech 26416-1-AP WB 1:10000
dsDNA Santa Cruz sc-58749 IF 1:250
Phosphor-STING (Ser366) Affinity AF7416 WB 1:2000
STING proteintech 19851-1-AP WB 1:2000
(Ser172) Affinity AF8190 WB 1:2000
TBK1 Santa Cruz sc-398366 WB 1:1000
Phosphor-IRF3 (Ser396) Affinity AF2436 WB 1:1500

	IRF3 Santa Cruz sc-33641 WB 1:1000<br>IFNA1 Affinity DF6086 WB 1:2000<br>IFNB Santa Cruz sc-57201 WB 1:1000
Validation	The antibodies were validated for the western blotting of both human and mouse samples on the websites of the associated companies (https://biorbyt.com.cn/, https://www.ptgcn.com/, https://www.cellsignal.cn/).

Eukaryotic cell lines

Policy information about cell lines and Sex and Gender in Research

Cell line source(s)	Primary cardiomyocytes (NRCMs)<br>Cardiac fibroblasts (NRFBs)
Authentication	Authentication of all the cell lines were performed by a Human STR Profiling Cell Authentication Service (ATCC).
Mycoplasma contamination	Cells tested negative for mycoplasma contamination.
Commonly misidentified lines<br>(See ICLAC register)	No misidentified lines were used in the study.

Animals and other research organisms

Policy information about studies involving animals; ARRIVE guidelines recommended for reporting animal research, and Sex and Gender in Research

Laboratory animals	Adult male C57BL6/N mice (Beijing Vital River Laboratory Animal Technology) aged 5-6 weeks were used in the study. The mice were housed under a 12-hour light/dark cycle at a temperature of 23 ± 1 °C and relative humidity of 50%-60%, with free access to water.
Wild animals	The study did not involve any wild animal.
Reporting on sex	The experiments in the study were done in male mcie. Relevant information is described detailly in the manuscript.
Field-collected samples	No field-collected samples were used in the study
Ethics oversight	All animal use and welfare adhered to the National Institutes of Health's Guide for the Care and Use of Laboratory Animals following a protocol reviewed and approved by the State Key Laboratory of Cardiovascular Disease, National Center for Cardiovascular Diseases, Fuwai Hospital (Beijing, China; permit number: 0000869). The study was reviewed and approved by the ethics committee of Fuwai Hospital (Bejing, China; No. FW-2019-0001).

Note that full information on the approval of the study protocol must also be provided in the manuscript.

Plants

Seed stocks	*Report on the source of all seed stocks or other plant material used. If applicable, state the seed stock centre and catalogue number. If plant specimens were collected from the field, describe the collection location, date and sampling procedures.*
Novel plant genotypes	*Describe the methods by which all novel plant genotypes were produced. This includes those generated by transgenic approaches, gene editing, chemical/radiation-based mutagenesis and hybridization. For transgenic lines, describe the transformation method, the number of independent lines analyzed and the generation upon which experiments were performed. For gene-edited lines, describe the editor used, the endogenous sequence targeted for editing, the targeting guide RNA sequence (if applicable) and how the editor was applied.*
Authentication	*Describe any authentication procedures for each seed stock used or novel genotype generated. Describe any experiments used to assess the effect of a mutation and, where applicable, how potential secondary effects (e.g. second site T-DNA insertions, mosiacism, off-target gene editing) were examined.*
